# Accurate characterization of the IFITM locus using MiSeq and PacBio sequencing shows genetic variation in Galliformes

**DOI:** 10.1186/s12864-017-3801-8

**Published:** 2017-05-30

**Authors:** Irene Bassano, Swee Hoe Ong, Nathan Lawless, Thomas Whitehead, Mark Fife, Paul Kellam

**Affiliations:** 1 The Wellcome Trust Sanger Institute, Wellcome Genome Campus, Hinxton, Cambridge, CB10 1SA UK; 2 Division of Infectious Diseases, Department of Medicine, Imperial College Faculty of Medicine, Wright Fleming Wing, St Mary’s Campus, Norfolk Place, London, W2 1PG UK; 3 The Pirbright Institute, Pirbright Laboratory, Ash Road, Woking, GU24 0NF UK

**Keywords:** PacBio RSII, Illumina MiSeq, Chicken IFITM, Genetic characterization, RNA-seq

## Abstract

**Background:**

Interferon inducible transmembrane (IFITM) proteins are effectors of the immune system widely characterized for their role in restricting infection by diverse enveloped and non-enveloped viruses. The chicken *IFITM* (*chIFITM*) genes are clustered on chromosome 5 and to date four genes have been annotated, namely *chIFITM1, chIFITM3, chIFITM5* and *chIFITM10*. However, due to poor assembly of this locus in the *Gallus Gallus* v4 genome, accurate characterization has so far proven problematic. Recently, a new chicken reference genome assembly *Gallus Gallus v5* was generated using Sanger, 454, Illumina and PacBio sequencing technologies identifying considerable differences in the chIFITM locus over the previous genome releases.

**Methods:**

We re-sequenced the locus using both Illumina MiSeq and PacBio RS II sequencing technologies and we mapped RNA-seq data from the European Nucleotide Archive (ENA) to this finalized chIFITM locus. Using SureSelect probes capture probes designed to the finalized chIFITM locus, we sequenced the locus of a different chicken breed, namely a White Leghorn, and a turkey.

**Results:**

We confirmed the Gallus Gallus v5 consensus except for two insertions of 5 and 1 base pair within the chIFITM3 and B4GALNT4 genes, respectively, and a single base pair deletion within the B4GALNT4 gene. The pull down revealed a single amino acid substitution of A63V in the CIL domain of IFITM2 compared to Red Jungle fowl and 13, 13 and 11 differences between IFITM1, 2 and 3 of chickens and turkeys, respectively. RNA-seq shows chIFITM2 and chIFITM3 expression in numerous tissue types of different chicken breeds and avian cell lines, while the expression of the putative chIFITM1 is limited to the testis, caecum and ileum tissues.

**Conclusions:**

Locus resequencing using these capture probes and RNA-seq based expression analysis will allow the further characterization of genetic diversity within Galliformes.

**Electronic supplementary material:**

The online version of this article (doi:10.1186/s12864-017-3801-8) contains supplementary material, which is available to authorized users.

## Background

Poultry accounts for almost half of all meat consumed in the UK, with 875 million chickens, 17 million turkeys, 16 million ducks and 250,000 geese a year supplied by over 2500 poultry farms [1]. Their production can be adversely affected by infection with avian specific viruses such as infectious bursal disease virus (IBDV), infectious bronchitis virus (IBV) and Newcastle virus (NDV) [2–7]. Poultry can also serve as the source of zoonotic, or potentially zoonotic, infections with viruses such as H5N1 and H7N9, transmitted to humans through contact with poultry. To reduce the threat to the global food supply and to minimize the risk of zoonotic events, there is an ongoing need to better understand the biology of avian viral infections, the mechanism of natural resistance (viral intrinsic and innate immunity) and the characterization of the biological factors that might be involved.

Interferon inducible transmembrane (IFITM) proteins are effectors of the immune system widely involved in restricting entry into cells of a broad range of viruses including Influenza viruses, Ebola and Zika [8–14]. In chickens, four *IFITM* genes have been annotated to date by the chicken gene nomenclature consortium (CGNC), namely *chIFITM1* (LOC422993-3-like), *chIFITM3* (LOC770612-1-like), *chIFITM5*, and *chIFITM10* [15–22]. Although not yet annotated by the CGNC, we have previously shown the existence of *chIFITM2* (putative LOC107053353-dispanin-2b-like) and suggested a hypothetical genetic structure of the locus based on the human syntenic genome region [23].

As IFN-stimulated genes (ISGs), *IFITM* abundance within a cell increases following activation of the type 1 IFN signaling pathway in response to the detection of pathogen associated molecular patterns (PAMPs) such as viral nucleic acid in the cytoplasm of the infected cell. In addition, binding of the IFNα/β to their cell surface receptors induces translocation of the transcription factor complex IFN-stimulated gene factor 3 (ISGF3) into the nucleus [24]. This induces the transcription of several ISGs, among which are the *IFITM* genes. The IFITM proteins target the final stages of viral entry by preventing fusion of the viral and cellular membranes [25]. This mechanism also reflects the localization of the human IFITM2 and IFITM3 which are found predominantly in intracellular membrane compartments such as late endosomes and lysosomes [21]. It is suggested that the membrane-defined site of fusion, namely plasma membrane and endosomes, is critical for the antiviral activity of these proteins [15].

While genetics and cell biology of the human IFITMs has been extensively characterized, lack of an accurate and complete reference genome sequence has hampered progress in characterizing the locus in diverse vertebrates including avian IFITMs. The genetic structure of the chicken locus was proposed based on the human locus however critical differences suggest that the current chIFITM nomenclature might be incorrect [23]. Indeed, the relative intracellular localizations of chIFITM1 and 2 as defined by genome synteny are the opposite of their human counterpart. This prompted Smith at al. to suggest an inversion might have occurred within the locus [23]. Subsequently, it was shown that duck IFITM1 localizes on the plasma membrane, like human IFITM1, highlighting further classification difficulty in avian IFITMs [16]. In addition, in the effort to explain the conserved antiviral activities of the different human *IFITMs* genes, Compton et al. have recently suggested that these differences, which reflect their localization and abundance in a cell, are a sign of a duplication and mutational events of the *IFITM* genes that arose millions of years [26]. Although their studies focused in the evolution of the *IFITM* genes in various non-human primates, it underlines the necessity to consider how avian *IFITM* genes should be considered as their nomenclature does not reflect necessarily their human orthologues. In this scenario, while ancestral IFITM3 is clearly syntenic with hIFITM3, more studies are required to elucidate the relationship between the other two IFITM proteins.

The most recent version of the chicken genome (v5) has incorporated long PacBio sequencing reads. This new sequencing has improved the chicken genome, including the IFITM locus. However, when sequencing an entire genome and performing whole genome assembly, minor assembly errors can occur, often due to lack of coverage or because paralogous sequences at other loci compromise accurate assembly. The IFITM gene family is one of the most paralogous families known with multiple copies of both IFITM genes and pseudogenes. For this reason, we sequenced just a small region of chromosome 5 containing the IFITM locus at high coverage with PacBio and with Illumina MiSeq*.*

The average PacBio read length is >10 kb, depending only on the activity of the polymerase [27–29] and although PacBio raw reads have a higher error rate compared to other technologies (14% versus 0.1 to 1% for Illumina), high quality consensus sequence can be obtained from overlapping reads. To complement the new *Gallus gallus* reference we have focused solely on the chIFITM locus and better elucidated its genetic structure by sequencing a bacterial artificial chromosome (BAC), from the BAC library used to generate the original *Gallus gallus* genome. The 203Kb-long BAC (CH261-109H20 [30]), containing the chIFITM locus, does not include chIFITM10. Given that the current literature focuses mainly on the antiviral activity of chIFITM1, 2, 3 and 5, we present evidence of the high confidence, high coverage sequence of this locus and the expression of these 4 genes by mapping of publicly-available RNA-seq data, to define each of the chIFITM proteins at the transcriptional level. Further, we describe the design and use of hybrid capture (SureSelect) probes and their use in genome capture and sequencing of other Galliform IFITM loci.

## Results

### *De novo* assembly of PacBio and MiSeq sequencing reads

**Fig. 1:**
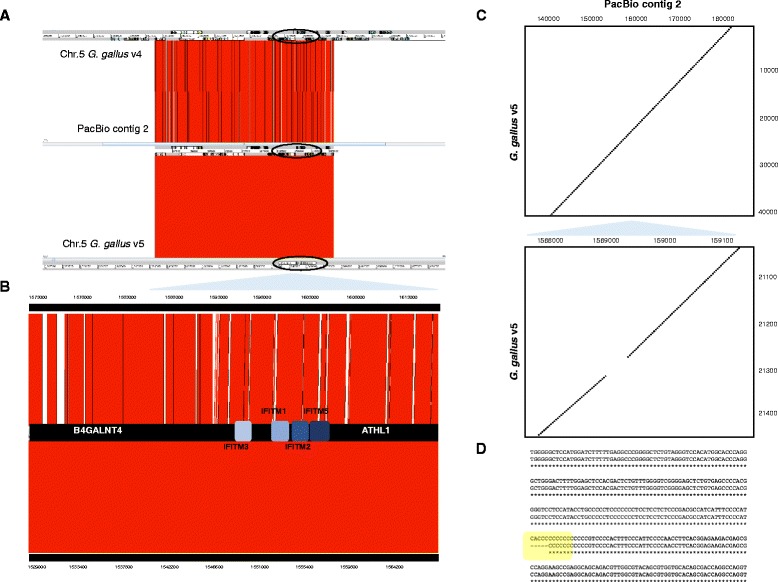
Locus comparison between PacBio consensus sequence (contig 2) and a portion of chromosome 5 of the two versions of the chicken genome. **a**: The 203 kb BAC reference sequence contained in the PacBio contig 2 (in the middle) is compared with chromosome 5 of *Gallus gallus* v4 (top) or v5 (bottom) using ACT, Artemis Comparison Tool. The annotation files for *Gallus gallus* v4 and PacBio contig 2 have been compressed to allow visualization of the whole BAC; for *Gallus gallus* v5 it was drawn manually only to visualize location of the locus. **b**: The chIFITM locus (circled in A) is enlarged in B to show only the chIFITM locus including the flanking genes (this is a 40 kb region extracted from the 203Kb total). Gaps are visible in *Gallus gallus* v4 represented by white bars (N nucleotides), while these are absent in the comparison with the more complete *Gallus gallus* v5. The graph does not show differences at the nucleotide level, but only an overall view of the locus. **c**: Dot Plot comparison graphs of the assembled PacBio contig 2 versus *Gallus gallus v5* showing differences not visible when using ACT for the 40Kb region. The region enlarged in the right Dot Plot shows a stretch of the genomic region within the intronic region of the *chIFITM3* gene which shows differences with chicken genome assembly v5. **d** Clustal Omega alignment of the PacBio contig 2 consensus sequences and the chicken genome v5 (portion of the IFITM3 gene corresponding to the gap seen in 2C). In yellow is highlighted the gap

In order to obtain a consensus reference sequence from the raw sequencing data, PacBio reads derived from the BAC clone sequencing were quality filtered and *de novo* assembled with HGAP using the protocols available on the SMRT portal (Additional file 1). Summaries of assembly and mapping statistics for PacBio (and also Illumina, see below) reads are shown in Table 1. Because of the length of the PacBio reads, the PacBio *de novo* assembled consisted of 6 assembled fragments (compared to 13 with Illumina). Of these, one contig (number 2, Table 2) contained the chicken genome sequence; the others represented genomic sequences from the *E.coli* BAC vector (Additional file 2). Contig 2, containing chicken sequences, had the highest base coverage and its length suggested it represented the full-length BAC clone. Therefore, to confirm the identity of this *de novo* assembled fragment, we utilized ACT and sequence similarly plots to compare contig 2 with chromosome 5 reference sequence from both *Gallus gallus* v4 and *Gallus gallus* v5 (Fig. 1a-c). Contig 2 contained the full chIFITM locus and highlights the substantial deficiencies to the *Gallus gallus* v4 genome assembly (Fig. 1a and b). This contrasts with *Gallus gallus* v5 genome assembly where fewer large gaps are observed, but with the presence of a small INDEL (Fig. 1c-d). Inspection of the similarity plot shows these differences observed at the nucleotide level fall in the genomic region of the *chIFITM3* gene, within the intronic region (Fig. 1c, bottom Dot Plot and sequence alignment in 1D). To further analyse this reagion we have also screened the full locus for repeats and low complexity DNA sequences as shown in Additional file 3. We attempted *de novo* assembly of Illumina MiSeq paired-end reads using three software packages (namely IVA, SGA and HGAP) resulting in only partial consensus sequence covering between 50 and 70% of the full chIFITM locus (including the flanking genes ATHL1 and B4GALNT4) (data not shown). The best assembly was generated using IVA, which produced the least number of contigs (13). In order to identify Illumina contigs that contained the BAC, and specifically the chIFITM locus, sequence similarity was used to compare the Illumina MiSeq contigs with the PacBio contig 2 (Additional file 4). All of the Illumina MiSeq contigs covered either portions of the PacBio contig 2, or just the chIFITM locus. These results suggest that while the longer PacBio reads map well to the reference genome (Additional file 5), Illumina MiSeq raw reads on their own are not be sufficient to assemble this region *de novo*, although they do map accurately to the *de novo* PacBio reference.

**Table 1:** PacBio and Illumina MiSeq de novo assembly and mapping statistics

	PacBio RSII		Illumina MiSeq	
Number of reads	78,140		665,450	
Number of Bases	401,758,407		199,635,000	
Mean Read Length	5141		300	
*De novo* assembly				
Assembly software	HGAP		IVA	
Polished contigs	6		13	
Sum of contigs length	4,818,915 bp		277,830 bp	
Largest fragment	2,323,934 bP		73,284 bp	
N50^a^	1,102,549 bp		NA	
Mapping				
Reference	Mapped reads	Mean coverage	Mapped reads	Mean coverage
Chr.5 *Gallus gallus* v4	33,892	193	586,297	607
Chr.5 *Gallus gallus* v5	34,068	196	693,474	440
PacBio_contig N.2	NA	NA	606,994	599

^a^N50 read length metric: The read length at which 50% of the bases are in reads longer than, or equal to, this value

**Table 2:** Basic statistic of *de novo* assembled contigs from PacBio reads

Contig	Length	Base called^a^	Consensus accuracy^b^	Base coverage^c^
1	2323934	1.0	0.99	38.8
2	223345	0.99	0.99	419.37
3	1102486	1.0	0.99	40.56
4	623652	0.99	0.99	38.0
5	537146	0.99	0.99	36.7
6	17862	1.0	0.99	28.56

^a^Bases Called: The percentage of reference sequence that has ≥ 1x coverage. % Bases Called + % Missing Bases should equal 100; ^b^Consensus Accuracy: The accuracy of the consensus sequence compared to the reference; ^c^Base Coverage: The mean depth of coverage across the reference sequence

### Organization of the chIFITM locus in PacBio contig 2 and *Gallus gallus* v4 and v5 reference sequences

**Fig. 2:**
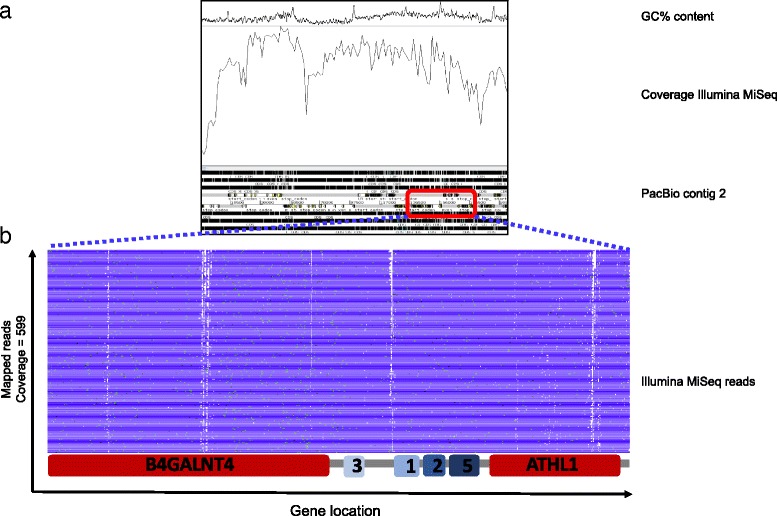
Artemis coverage and stack view of Illumina MiSeq reads mapped against PacBio consensus sequence (contig 2). **a** Overall coverage and GC content of the Illumina MiSeq BAC reads (203 kb region) mapped against the PacBio contig 2. This reference was built using the annotation of *Gallus gallus* v4 as scaffold. The *chIFITM* genes are located between 138150 and 177724 in the 203Kb region. **b** stack view of the Illumina MiSeq reads showing the chIFITM locus

To study in more detail the gene order of the v4 and v5 assembled locus relative to our assembly we used Artemis. Concentrating on the *chIFITM* genes, we show that combined reads from both sequencing technologies mapped well to v4 or v5 assemblies, covering the locus to significant depths and aligning to all the regions of interest (Fig. 2 and Additional file 5A-D). The deep and accurate sequence of the chIFITM locus allows us to be confident that the chIFITM1 and 2 genes as named and annotated in the v5 genome are indeed inverted in comparison to the human locus with chIFITM1, 2, 3 and 5 genes having their transcriptional units in the same direction (Table 3) [23].

**Table 3:** Coordinates of the *chIFITM* genes within the PacBio consensus sequence (contig 2)

Gene	Location in contig 2
chIFITM1	162068..163611
chIFITM2	164151..165395
chIFITM3	158589..159917
chIFITM5	165955..167524
ATHL1	168807..177724
B4GALNT4	138150..157395

### SureSelect probes design and pull down of the IFITM locus from turkey breast tissue and DF1 cells

**Fig. 3:**
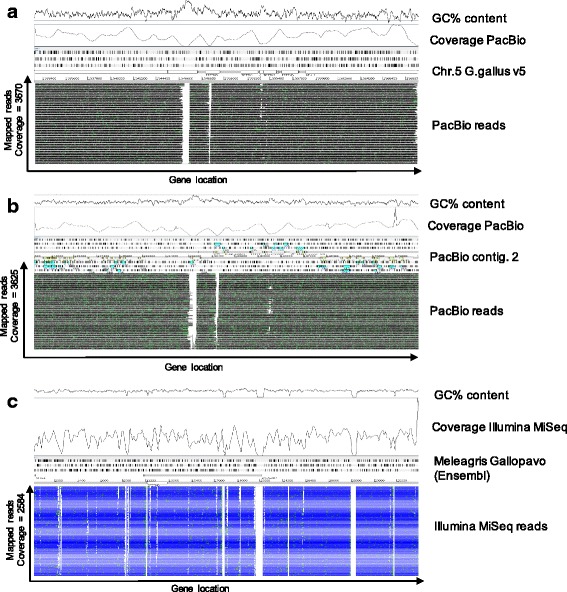
**a** Artemis coverage and stack view of the IFITM locus in DF1 cells following pull down of the IFITM locus using SureSelect probes and sequencing with PacBio. The figure shows an intact locus and successful mapping of the IFITM locus against the Gallus *gallus* sequence reference, despite two gaps observed within the *B4GALNT4* and *IFITM3* genes. **b** Artemis coverage and stack view of the IFITM locus in DF1 cells following pull down of the IFITM locus using SureSelect probes and sequencing with PacBio. These reads were instead mapped against the new PacBio contig 2 sequence reference. As for the mapping above, two gaps (one partial) are observed within the *B4GALNT4* and *IFITM3* genes, although more reads cross the gaps, allowing full coverage. **c** Artemis coverage and stack view of the IFITM locus in turkey breast tissue following pull down of the IFITM locus using SureSelect probes and sequencing with Illumina MiSeq. The graph shows successful mapping of MiSeq reads despite using chicken probes to pull down the locus in turkey tissue. The white bars represent actual gaps in the turkey reference as published on both Ensemble and NCBI and to which the probes will not eventually map as gaps are shown in the reference as “NNN”

**Fig. 4:**
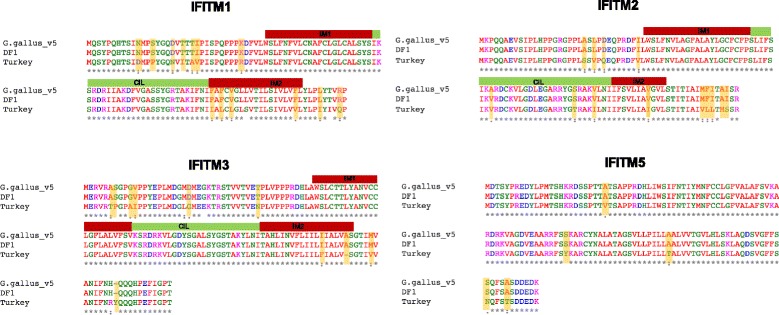
Clustal Omega alignment of the amino acid sequence of the IFITM proteins derived from the consensus sequence of DF1 and turkey samples following targeted SureSelect pulldown. The amino acid sequences are compared to the *Gallus gallus* v5 sequences. Domain structures are represented as: IM1 and IM2, intramembrane domain 1 and 2, CIL, conserved intracellular loop. These have yet to be defined for chIFITM5

The consensus sequence we have generated was used to design Agilent SureSelect probes covering the 40 kb region encompassing the IFITM locus. Our primary purpose is to use these probes to study possible IFITM variants in different chicken breeds and further into the phylogeny of Galliformes. We were able to successfully pull down the IFITM locus in DF1 cells (chicken embryonic fibroblasts) as well as turkey breast tissue (Fig. 3), showing we are able to use chicken (Phasianinae, sub-family of Galliformes) IFITM probes to pulldown and sequence the locus in a different Galliform sub-family, namely the Meleagridinae, to which the turkey belongs. The BAC clone, like *Gallus gallus* v5 of the chicken genome, is from a Red Jungle fowl, inbred line UCD001 (Inbred 256, female) while the DF1 cells are derived from a White Leghorn (East Lansing line-0, 10-day old eggs). Mapping of PacBio reads from DF1 cells against either v5 of the chicken genome sequence or our PacBio contig 2 gives a good coverage but with low coverage gaps detected in IFITM3 and B4GALNT4 (Fig. 3a-b). The IFITM3 gap was closable with the low frequency PacBio reads and the PacBio contig 2 reference, yielding an accurate IFITM locus sequence for DF1 cells. Illumina sequencing of the turkey IFITM locus assembles more poorly to the turkey reference genome (Fig. 3c), suggesting the current turkey genome is in need of improvement with long read PacBio sequences as achieved for the chicken genome. We were however, able to identify all four IFITM genes in the turkey locus. We constructed multiple sequence alignments for the two chicken and turkey genome IFITMs (Fig. 4). Amino acid sequence alignment of the IFITM proteins of DF1, turkey and *Gallus gallus* v5 shows substantial differences as we can see from Fig. 4. For the known antiviral IFITMs one amino acid change was found between Red Jungle fowl and White Leghorn, namely A63V in the CIL domain of IFITM2. More amino acid substitutions were seen for Turkey compared to chicken IFITMs with 13, 13 and 11 differences between IFITM1, 2 and 3 respectively. Variation in one of the chicken IFITMs is maintained in the turkey gene, namely amino acid 63 A (Red Jungle Fowl) or V (White Leghorn) and 63 V (Turkey) in IFITM2.

### Mapping RNA-seq data to the PacBio contig 2 reference containing the chIFITM locus

The generation of a high quality *de novo* assembly of the IFITM locus sequence allows accurate mapping of RNA-sequence data from previous published studies for qualitative and quantitative analysis. To validate which chIFITM transcripts were expressed, and to assess their level of expression, we first used RNA-seq reads from 293 T cells, engineered to express only chicken IFITM proteins constitutively. Reads from the control cells (wild type 293 T) do not map to the chIFITM locus (Table 4). Focusing on the 40 kb region containing the chIFITM locus, including the flanking genes *ATHL1* and *B4GALNT4*, we observed RNA-seq reads from 293 T cell lines stably expressing chIFITM1, 2, or 3 with expected peaks of expression at gene exon locations (Additional file 6). The number of mapped reads and by implication the expression level for chIFITM3 was higher than that of chIFITM2 and in turn both higher than that of chIFITM1 (Additional file 6). We analysed 26 RNA-seq studies totaling 293 sequenced chicken tissues and avian cell lines that were identified in the ENA database. The samples were examined for constitutive expression levels of the chIFITMs in a subset of each study covering at least one immune relevant tissue type (Table 5). To analyze constitutive expression, RNA-seq data from liver, spleen, lung and trachea samples taken from the studies as listed in Table 6, were mapped against the PacBio contig 2. To these, we added expression data from commonly used laboratory cell lines (DF1, CEF, HD11, DT40).

**Table 4:** chIFITM transcripts average coverage values in the stable cell lines

Cell line	Average coverage
293 T	35
293 T - chIFITM1	34
293 T - chIFITM2	339
293 T - chIFITM3	746

**Table 5:**
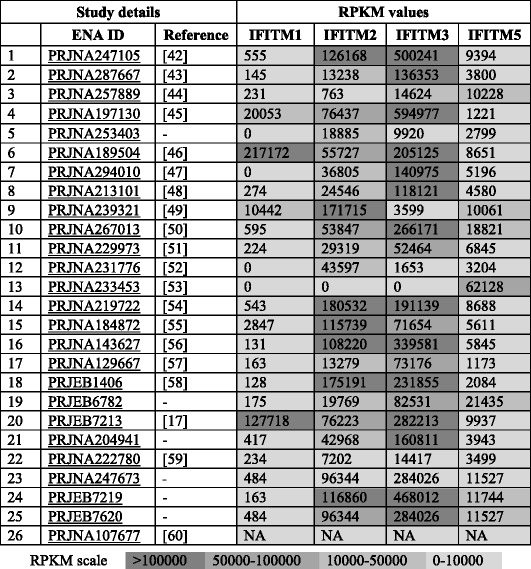
Expression levels of the IFITM transcripts calculated as RPKM in the different RNA-seq studies deposited in the European Nucleotide Archive (ENA) database

RPKM values were calculated for all the samples present in each study by Artemis. (*NA: BWA did not detect any BAM alignment across the reference provided.*) [17, 42–60]

**Table 6:** Tissue types, experimental conditions and species considered in the different RNA-seq studies

N.	Tissue	Condition	Species
1	Lung	H5N3 AIV	Fayoumi and leghorn
2	DF-1	IRF 7 overexpression and knockdown assays/poly I:C	East Lansing Line (ELL-0) White Leghorn
3	DF-1	Cell-adapted Infectious Bursal Disease Virus (ca-IBDV) infection	East Lansing Line (ELL-0) White Leghorn
4	Trachea	Infectious laryngotracheitis virus vaccine	15-day-old SPF white leghorn chickens
5	DT40 CL18 chicken B lymphoma cells	Basal	Bursal lymphoma cell line derived from a Hyline SC chicken
6	Caecal tissue	C.*jejuni* strain NCTC11168v1	Barred Rock chickens
7	Breast muscle	Basal	White rock/Xinghua chickens
8	Abdominal adipose tissue	Body weight	7 week old broiler chickens
9	Primary hepatocellular carcinoma epithelial cell line	Heat stress response	Chicken male white-leghorn hepat ocellular (LMH) cell line.
10	Spleen	J Subgroup Avian Leukosis Virus (ALV-J) Infection	White Recessive Rock
11	Facial	Talpid2 heterozygous carriers	HH25 chickens
12	DT40 cells	Splicing factor SRSF10	Bursal lymphoma cell line derived from a Hyline SC chicken
13	MSB1 cell line	Marek’s disease virus 1	Chicken lymphoblastoid cell line
14	Liver	Heat stress response	Broiler chickens
15	Endocardial cells	Endocardial EMT	HH18 chicken/embryo
16	Brain (cerebral cortex/whole brain without cerebellum), cerebellum, heart, kidney, liver and testis	Basal	Red jungle fowl
17	Liver/muscle	Basal	7 day red jungle fowl and broiler
18	CEF/HD11	Lipopolysaccharide	11-day white leghorn
19	Mid shaft tibial bone	Basal	White leghorn
20	Ileum/lung	H5N2/H5N1	White leghorn/Domestic Gray Mallards
21	Adrenal gland, adipose, cerebellum, testis, ovary, heart, hypothalamus, kidney, liver, lung, breast muscle, sciatic nerve, proventriculus, spleen	Basal	Red Jungle Fowl
22	Whole embryo	Basal	UE1295 PEAT/F-37380 cross
23	Testis		New Hampshire
24	Spleen	IBDV	Gallus gallus
25	CEF	chIFNα	CEF
26	Chicken embryo	Basal	Gallus Gallus

**Fig. 5:**
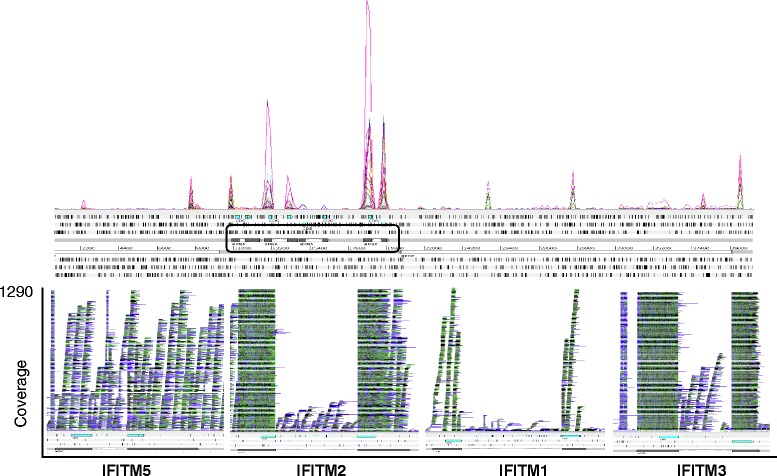
The read alignment views in Artemis showing RNA-Seq data from the different studies. Top panel: the ‘coverage view” showing a separate plot for each BAM mapped to our PacBio contig 2 (40Kb region). The coverage shows only data relative to constitutive expression level of chIFITMs in immune-relevant tissues and cell lines (lung, trachea, spleen, liver, DF1, CEF, HD11, DT40). Bottom panel: the “stack view” (paired reads: blue, single reads and/or reads with an unmapped pair: black; reads spanning the same region: green) to show in more detail read depth across each chIFITM transcript. All the features were annotated manually blasting the sequences from the latest version of the chicken genome. Cyan: CDS region, grey: mRNA, white: gene (overlapping with mRNA features)

**Fig. 6:**
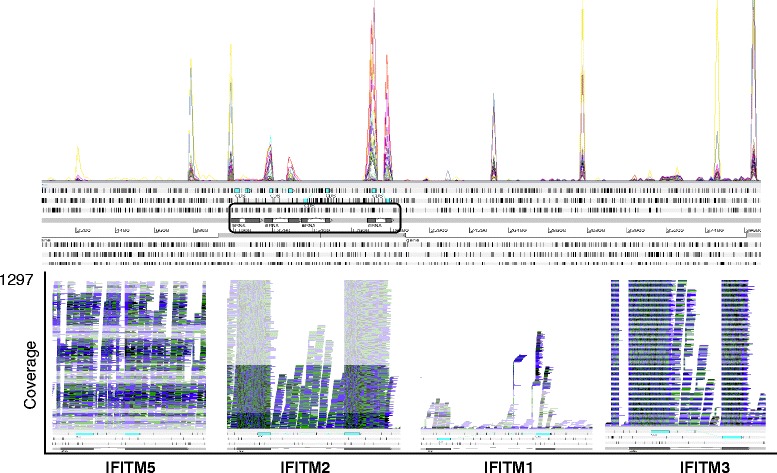
RNA-seq data alignment of reads from the immune relevant tissues and cell lines in treated conditions: infection with IBDV, ALV-J, ILVV, LPS, H5N5/H5N1 or heat stress-induced conditions. The graph shows that also in these conditions, levels of chIFITM are lower compared to chIFITM2 and chIFITM3. Top panel, overall coverage. Bottom panel stack view of each chIFITM transcript

ChIFITM3 is constitutively expressed (both exons) in all tissues and cell lines analysed at levels higher than the putative chIFITM1 and chIFITM2. Indeed, putative chIFITM1 is barely detectable in most of the tissues, and much lower compared to the other IFITM transcripts, as also shown from the RPKM values in Table 5. Further, when infected or subject to cellular stress chIFITM2 and chIFITM3 are abundantly expressed, again with little IFITM1 expression. Indeed, it is not possible to detect convincing levels of IFITM1 expression at any time except for Caecal tissue and Ileum tissue infected by influenza A H5N2 or H5N11 (Figs. 5 and 6, Additional file 7 and Table 5). In addition, the coverage graphs confirm that the typical genetic structure of the *chIFITM* genes is maintained, with two exons separated by a single intron in all cases, although reads were observed to map beyond the boundaries of the annotated genes particularly in the stretch of genomic region between IFITM2 and 5 (Figs. 5 and 6).

## Discussion

In this study we have sequenced a BAC clone containing the complete chIFITM locus using both PacBio and Illumina MiSeq sequencing technologies producing an accurate assembly of the locus. We analysed expression levels of the *chIFITM* genes using publicly available RNA-seq data from different chicken lines and tissues, and produced hybrid capture probes for ‘pull-down’ sequencing of another chicken line and the more distant turkey IFITM locus.

The chIFITM locus showed several gaps in the version 4 of the chicken genome release (*Gallus gallus* 4). It had been improved by sequencing the same DNA reference source (Female Red Jungle Fowl, UCD001 inbred line) with PacBio technology. Comparison of the two public versions of the chIFITM locus with the one generated in our study (PacBio contig 2) still demonstrated differences, despite being the same inbred line. We believe these discrepancies in the public genome assemblies might be a consequence of genome wide assembly required for full chicken genome, suggesting that our BAC sequence (203 kb) is likely to be more accurate, particularly in GC-rich regions. In addition, quality control analysis and type of assembler used will influence the final consensus sequence generated for any region of the chicken genome, leading to the differences observed in the sequences. To produce our sequence, we employed both PacBio RSII and Illumina MiSeq technologies because they have complementary properties that met our requirements for covering gaps and maintaining sequence integrity. Sequencing within *Gallus gallus domesticus* lines, more outbred chickens and more divergent Galliforms is now possible using hybrid capture genome sequencing. Indeed, we have been able to document many amino acid sequence changes between chickens and turkeys in the antiviral IFITMs in regions of the proteins known to be important for their antiviral activity (Fig. 4).

The importance of obtaining an accurate sequence is vital to understand the genetic structure and confirm the identity of the *IFITM* locus, thus to correctly annotate the genes. Hypothetical structures of the chIFITM locus have been suggested, based on the human locus but inconsistencies remain between alignments for the putative *chIFITM1* and *chIFITM2* [16, 17, 23]. Based on the literature and current annotation the four genes are clustered on chromosome 5 which also contains the *chIFITM10* gene (the function of which remains to be elucidated). Following the discovery of *chIFITM2,* Smith at al. [23] proposed an organizational structure for the locus, based on features such as membrane localization and lack of an N-terminal extension (both characteristic of the IFITM2 and IFITM3 proteins), suggesting that chIFITM2 is actually analogous to human IFITM1 [23]. Our immunofluorescence analysis to study localization of the chicken proteins expressed in human (293 T) stable cell lines is in agreement with Smith et al. (data not shown, *Bassano* et al. *in preparation*) [23]. Indeed, chIFITM2 is membrane-bound, while chIFITM1 localizes to the early endosomes. Here our RNA-seq analysis of the ENA dataset shows that *chIFITM1* basal expression levels are very low compared to *chIFITM2* and *chIFITM3*. The analysis of the samples in presence of IFN$\boldsymbol{\upalpha}$, H5N2, H5N1, H5N3, IBDV, IRF7, ALV, Lipopolysaccharide or in heat-stress induced conditions, also shows that higher expression levels can be observed for chIFITM3 and chIFITM2 suggesting a key role for these two proteins as antiviral IFITMs compared to chIFITM1, expression of which is only in the intestinal tract and in the testis. Although immunofluorescence staining seems to suggest that chIFITM2 is analogous to hIFITM1 (they are both plasma membrane-bound) the genome organisation supported by long read PacBio sequences now unambiguously confirms that the chIFITM2 and chIFITM1 locus is inverted compared to the human locus. We therefore, propose based on gene expression, genome architecture and published functional data the gene order in the chicken locus on chromosome 5q should be renamed: centromeric – *B4GALNT4 – chIFITM3 – chIFITM2 – chIFITM1 – chIFITM5 – ATHL1* – telomeric.

## Conclusions

In this report we have produced an updated genomic map of chIFITM locus that includes the two flanking genes *ATHL1* and *B4GALNT4*, by combining and analyzing sequencing data derived from PacBio RS II and Illumina MiSeq sequencing technologies. The only difference detected in our assembled locus sequence relative to the *Gallus Gallus (v5)* is a 5 bp insertion in the intronic region of *chIFITM3*. This change in sequence may not have any influence on the function and expression of the *chIFITM3* gene. However, RNA-seq analysis shows expression of all IFITMs from this locus but that *chIFITM1* has different patterns of expression from the other antiviral IFITMs. Initial analysis of different chicken breeds shows IFITM amino acid variation between different chicken breeds and turkeys.

## Methods

### Bacterial Artificial Chromosome (BAC) construct recovery

The BAC clone (CHORI-261) from Red Jungle Fowl strain UCD001 covering the predicted IFITM locus was purchased from BACPAC Resources Centre. The BAC clone, delivered as a stab culture was streaked directly on Luria Broth (LB) agar (chloramphenicol 12.5 μg/mL) to isolate single colonies and incubated overnight at the designated growth temperature. Single colonies were picked and cultured in LB media. Plasmid DNA was then extracted and purified according to Qiagen Plasmid DNA kit manufacturer’s protocol.

### Sequencing, assembly and alignment

A total of 3 μg isolated plasmid DNA was sequenced across two platforms, the Illumina MiSeq and PacBio RSII. Library preparation and quality control was undertaken by The Wellcome Trust Sanger Institute’s core sequencing facility. Assembly of PacBio sequencing reads was performed using protocols available on the SMRT^®^ Portal. Briefly, sequencing fragments were first filtered to remove reads that did not meet read quality and length thresholds, then *de novo* assembled using HGAP [31]. Errors in the re-circularization of the BAC as well as sequence consensus generation for the DF1 cell line were corrected using iCORN v2, Interative Correction of Reference Nucleotides [32]. MiSeq reads were first analysed for low quality reads with FastQC [33] and low quality reads were trimmed using Trimmomatic [34]. *De novo* assembly of MiSeq reads was attempted using IVA [35], SGA [36] and HGAP from the SMRT^®^ Analysis package [31]. SMALT, (http://www.sanger.ac.uk/science/tools/smalt-0) a pairwise sequence alignment program was used to map MiSeq reads onto genomic reference sequences, either chromosome 5 of *Gallus gallus* (v4 and v5) or the consensus sequence generated from *de novo* assembly of PacBio sequencing reads. The SAM files generated were converted into indexed BAM files using Samtools 0.1.19 [37]. Artemis (v13.0) and ACT (Artemis Comparison Tool) [38] were used to analyse locus coverage and accuracy of the alignment. Comparison files required to run ACT were generated with megablast [39]. Dot plots were generated calling dotter from the command line [40]. Annotation for the PacBio consensus sequence was generated by RATT, Rapid Annotation Transfer Tool [41] using as scaffold the annotation from *Gallus gallus* version 4.

All sequences produced in this manuscript are deposited in the ENA under the accession numbers ERS556272, ERS565108, ERS1276179, PRJNA361311.

### SureSelect pull down of the IFITM locus

SureSelect probes covering the chicken IFITM locus (40Kb region) were purchased from Agilent and samples processed for targeting pulldown according to the Illumina and PacBio protocols.

### Cell culture

Two hundred ninety-three T and DF1 cells were cultured in DMEM medium supplemented with 10% FCS, in absence of any antibiotics. Stable transfections were performed using Fugene (Promega) according to the manufacturer’s instructions and cells maintained in culture in presence of puromycin for positive selection. RNA extraction was performed using Qiagen RNA extraction kit according to the manufacturer’s instructions. Up to 5 μg of extracted RNA was reverse transcribed and sequenced using Illumina MiSeq. DNA extraction from turkey breast tissue was performed using Qiagen Tissue and blood DNA extraction kit, according to the manufacturer’s protocol.

### European Nucleotide Archive (ENA) sequencing data download and RNA-seq analysis

RNA-seq datasets for this study were retrieved from ENA records (Table 5). A total of 26 studies for chicken sequencing datasets were identified. FastQC-corrected reads were aligned to the PacBio-derived consensus sequence using BWA version 0.7.12-r1039, Samtools 0.1.19 and MAFFT version 7.205. The BAM files generated were then visualized using Artemis. To quantify transcripts expression, RPKM (Reads Per Kilobase per Million mapped reads) were calculated using Artemis by selecting the feature of interest. Read depth for RNA-seq alignment was calculated using Ugene v1.25.0.

## Additional files

Additional file 1: Schematic representation of the PacBio and Illumina MiSeq sequencing pipelines. Samples are first sheared and libraries created for Illumina or PacBio following specific protocols. Although many of the steps are shared between the two technologies, PacBio does not involve a PCR step before sequencing, characteristic of all the Illumina sequencing protocols. (PDF 43 kb)

Additional file 2: Dot plot showing sequence comparison between *E.coli* genome and the contigs obtained from *de novo* assembly of PacBio sequencing reads. As shown in the graph, one contig (2), does not align to the bacterial genome, being the chicken sequence within the BAC clone. (PDF 33 kb)

Additional file 3: Analysis of repeat elements along the chicken IFITM locus on chromosome 5. RepeatMasker was ran along the 40 kb region using the UCSC genome browser platform. The figure shows Short interspersed nuclear elements (SINE), which include ALUs, Long interspersed nuclear elements (LINE), Long terminal repeat elements (LTR), which include retroposons, DNA repeat elements (DNA), Simple repeats (micro-satellites), Low complexity repeats, Satellite repeats, RNA repeats (including RNA, tRNA, rRNA, snRNA, scRNA, srpRNA), Other repeats, which includes class RC (Rolling Circle). Shades of the repeats reflect the amount of base mismatch, base deletion, and base insertion associated with a repeat element. The higher the combined number of these, the lighter the shading. In red is shown the gene annotation (Ensemble), however, this has not been updated, since IFITM2 has not been annotated yet. (PDF 555 kb)

Additional file 4: Dot Plot of Illumina MiSeq IVA contigs versus PacBio contig 2. The 13 IVA *de novo* assembled MiSeq contigs (separated by vertical green lines) are plotted against PacBio contig 2 to identify the contig that covers the BAC or the chIFITM locus. The chIFITM locus including the flanking genes within contig 2 is shaded in blue showing that none of the contigs fully covers the region of interest. (PDF 29 kb)

Additional file 5: Alignment of PacBio and MiSeq reads against *Gallus gallus* v4 (left panel) and *Gallus gallus* v5 (right panel). A.: Artemis coverage view of the reads, blue = PacBio, red = MiSeq, black = GC content of the reference. B/C/D: Artemis “stack” view of the chIFITM locus; B shows mapping of MiSeq reads, C mapping of PacBio reads and D the overlapped alignment of PacBio and MiSeq reads. (PDF 1860 kb)

Additional file 6: RNA-seq data mapping of 293 T cells-derived reads to the consensus sequence obtained from PacBio sequencing (contig 2). A: Mapping of reads from not transfected 293 T cells. B/C/D.: Mapping of 293 T cells stably expressing chIFITM3, 2, 1, respectively. The figure only shows a detail of the locus, encompassing the three main genes. (PDF 561 kb)

Additional file 7: A and B: RNA-seq data alignment of reads from caecal and ileum tissues (A) and testis (B) showing high coverage chIFITM1, not seen in the other studies analysed. C: RNA-seq data alignment of reads from bone tissue showing high coverage for chIFITM5. The figure focuses only on the 4 chIFITM transcripts showing on the left panel the coverage and on the right panel the stack view. The stack view of A and B also shows some distinct coverage for chIFITM5, more ordered than the other studies. (PDF 763 kb)
