# Supplementary figures and images for: Accurate characterization of the IFITM locus using MiSeq and PacBio sequencing shows genetic variation in Galliformes
Source: BMC Genomics. 2017 May 30;18:419. doi: 10.1186/s12864-017-3801-8 (PMC5450142; doi:10.1186/s12864-017-3801-8)

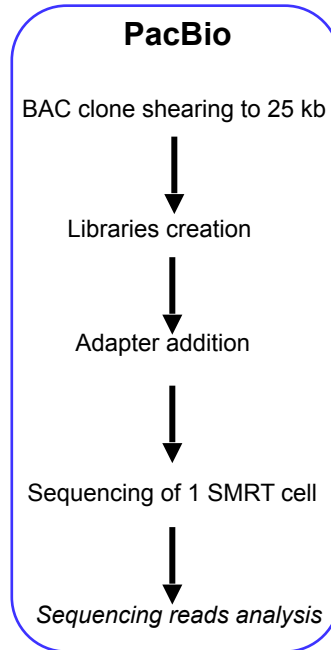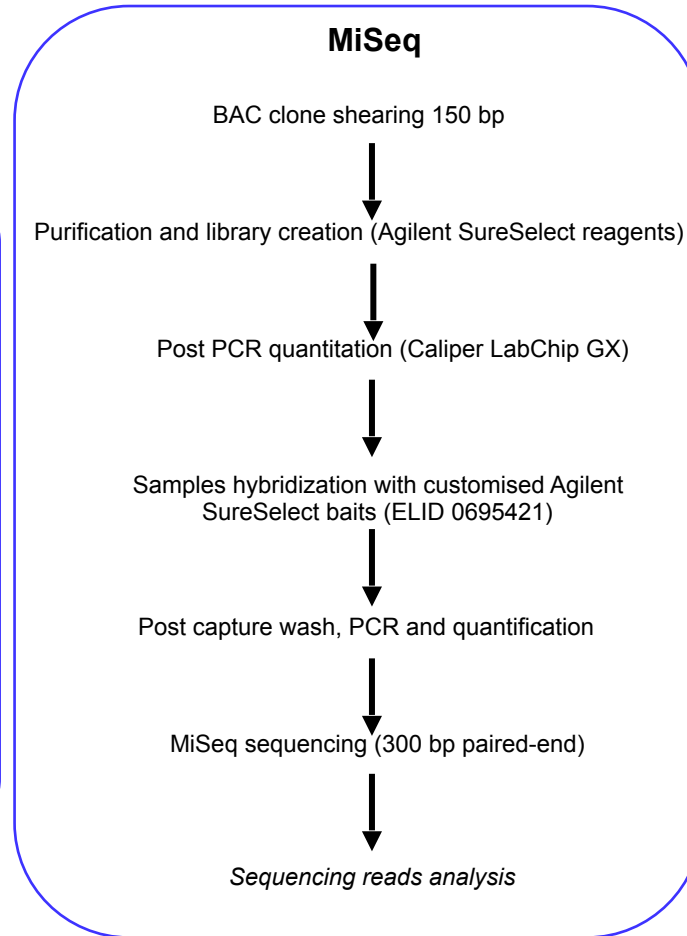

Supplement: Supplementary file 1 — Schematic representation of the PacBio and Illumina MiSeq sequencing pipelines. Samples are first sheared and libraries created for Illumina or PacBio following specific protocols. Although many of the steps are shared between the two technologies, PacBio does not involve a PCR step before sequencing, characteristic of all the Illumina sequencing protocols. (PDF 43 kb) [file 12864_2017_3801_MOESM1_ESM.pdf]

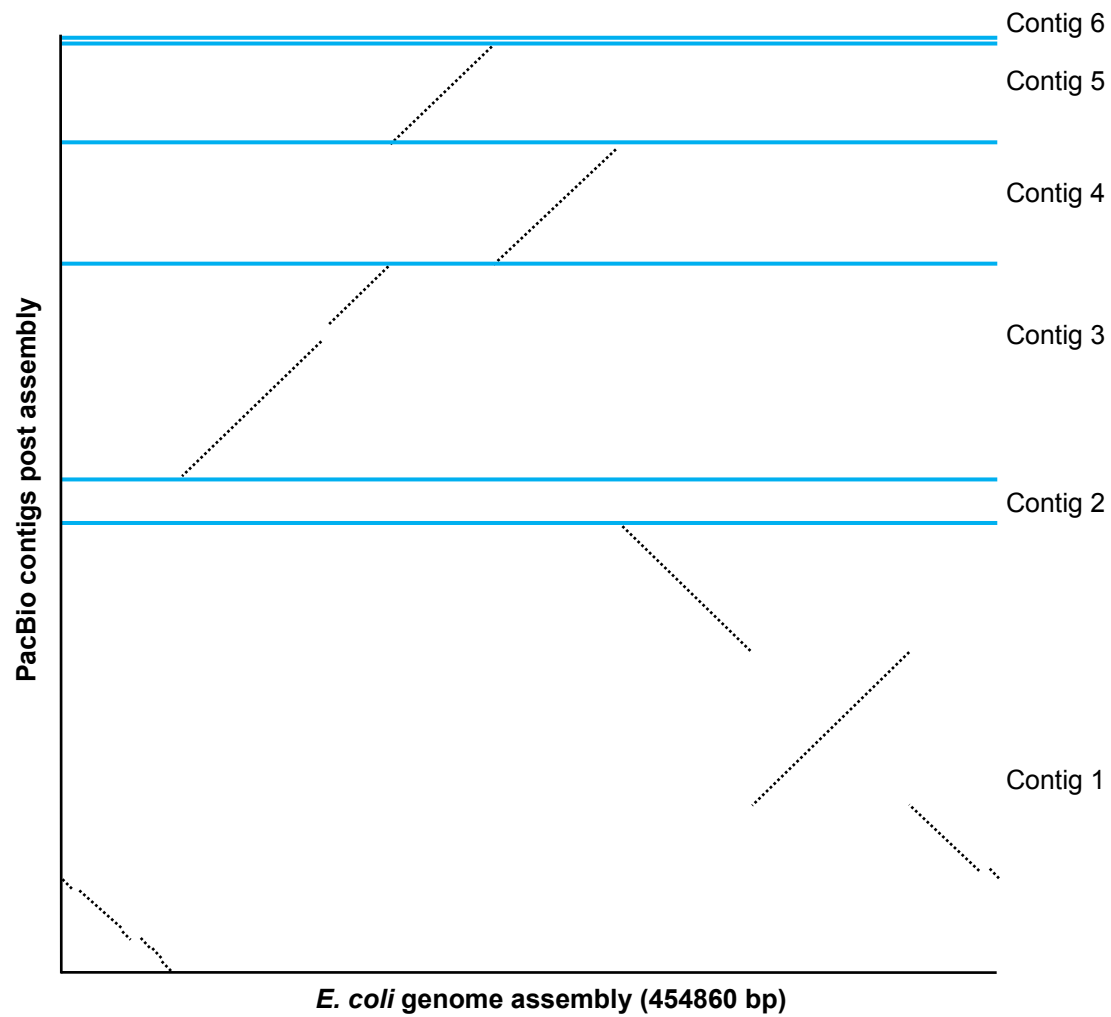

Supplement: Supplementary file 2 — Dot plot showing sequence comparison between E.coli genome and the contigs obtained from de novo assembly of PacBio sequencing reads. As shown in the graph, one contig (2), does not align to the bacterial genome, being the chicken sequence within the BAC clone. (PDF 33 kb) [file 12864_2017_3801_MOESM2_ESM.pdf]

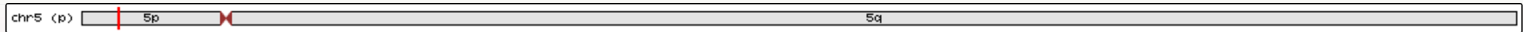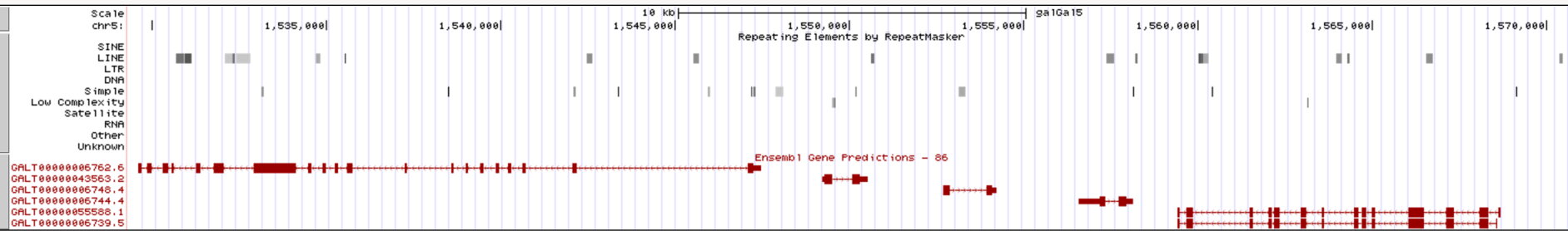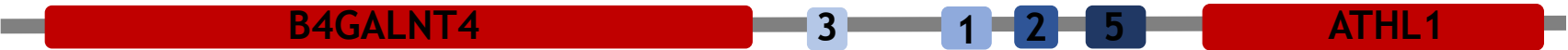

Supplement: Supplementary file 3 — Analysis of repeat elements along the chicken IFITM locus on chromosome 5. RepeatMasker was ran along the 40 kb region using the UCSC genome browser platform. The figure shows Short interspersed nuclear elements (SINE), which include ALUs, Long interspersed nuclear elements (LINE), Long terminal repeat elements (LTR), which include retroposons, DNA repeat elements (DNA), Simple repeats (micro-satellites), Low complexity repeats, Satellite repeats, RNA repeats (including RNA, tRNA, rRNA, snRNA, scRNA, srpRNA), Other repeats, which includes class RC (Rolling Circle). Shades of the repeats reflect the amount of base mismatch, base deletion, and base insertion associated with a repeat element. The higher the combined number of these, the lighter the shading. In red is shown the gene annotation (Ensemble), however, this has not been updated, since IFITM2 has not been annotated yet. (PDF 555 kb) [file 12864_2017_3801_MOESM3_ESM.pdf]

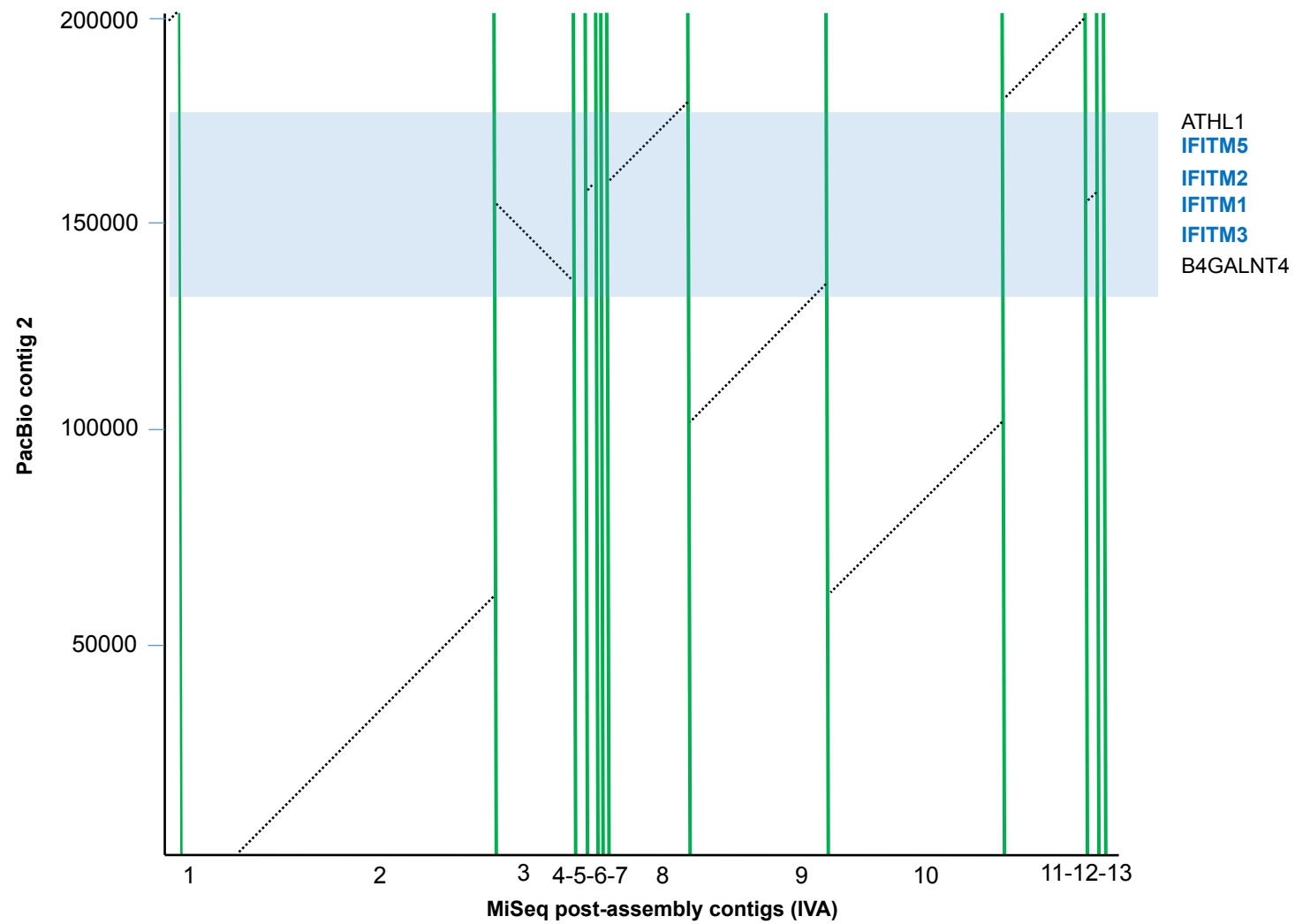

Supplement: Supplementary file 4 — Dot Plot of Illumina MiSeq IVA contigs versus PacBio contig 2. The 13 IVA de novo assembled MiSeq contigs (separated by vertical green lines) are plotted against PacBio contig 2 to identify the contig that covers the BAC or the chIFITM locus. The chIFITM locus including the flanking genes within contig 2 is shaded in blue showing that none of the contigs fully covers the region of interest. (PDF 29 kb) [file 12864_2017_3801_MOESM4_ESM.pdf]

# *G. gallus* v4

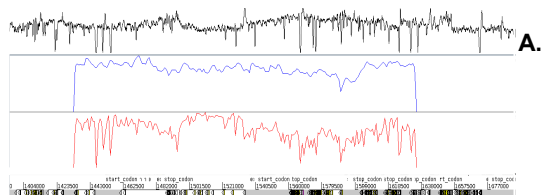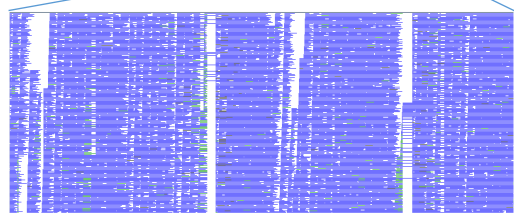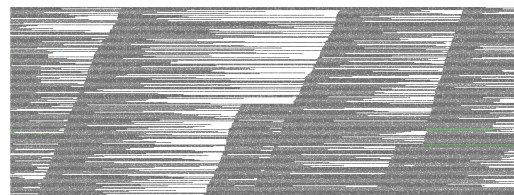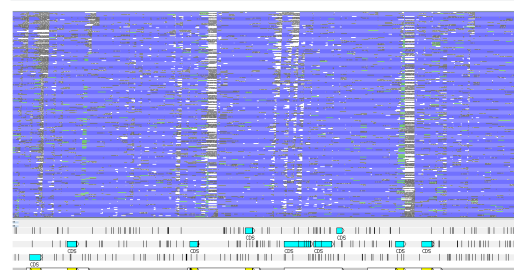

# *G. gallus* v5

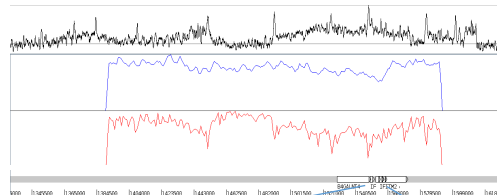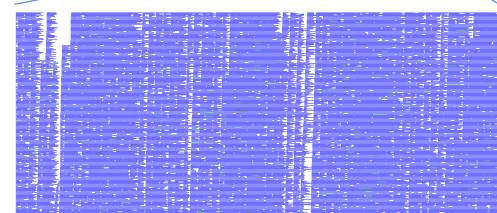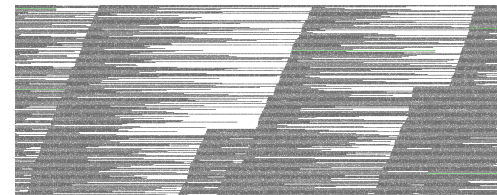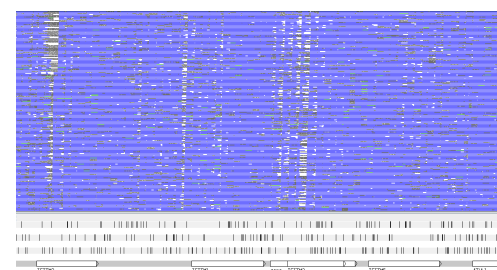

Supplement: Supplementary file 5 — Alignment of PacBio and MiSeq reads against Gallus gallus v4 (left panel) and Gallus gallus v5 (right panel). A.: Artemis coverage view of the reads, blue = PacBio, red = MiSeq, black = GC content of the reference. B/C/D: Artemis “stack” view of the chIFITM locus; B shows mapping of MiSeq reads, C mapping of PacBio reads and D the overlapped alignment of PacBio and MiSeq reads. (PDF 1860 kb) [file 12864_2017_3801_MOESM5_ESM.pdf]

**293T**

**293T-chIFITM1**

**A.**

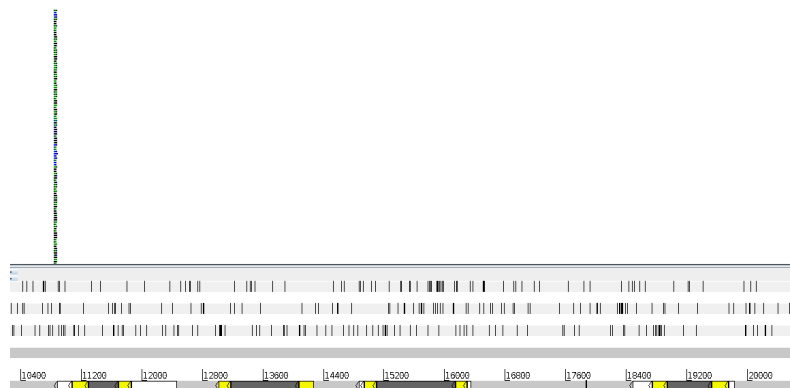

**B.**

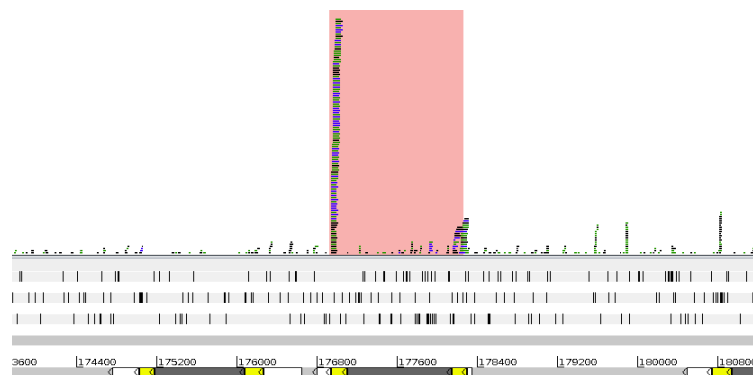

**293T-chIFITM2**

**293T-chIFITM3**

**C.**

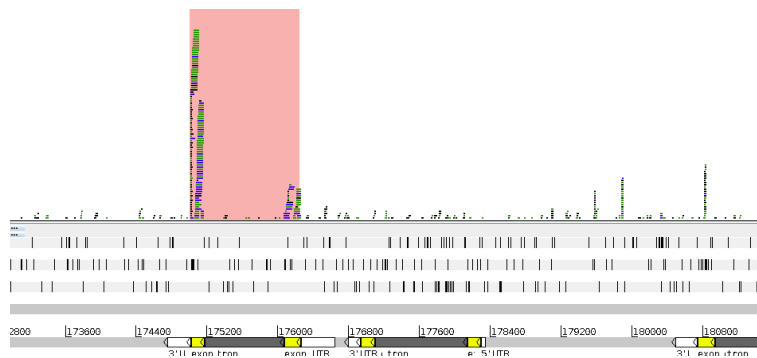

**D.**

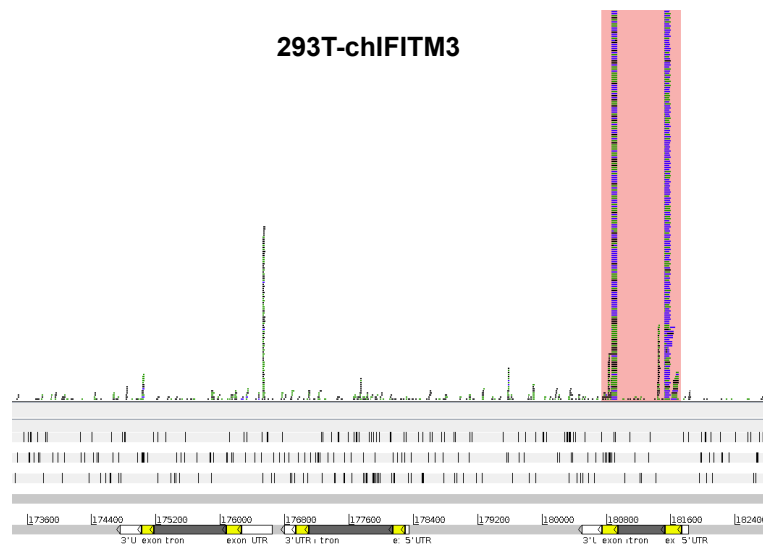

Supplement: Supplementary file 6 — RNA-seq data mapping of 293 T cells-derived reads to the consensus sequence obtained from PacBio sequencing (contig 2). A: Mapping of reads from not transfected 293 T cells. B/C/D.: Mapping of 293 T cells stably expressing chIFITM3, 2, 1, respectively. The figure only shows a detail of the locus, encompassing the three main genes. (PDF 561 kb) [file 12864_2017_3801_MOESM6_ESM.pdf]

### Caecal and ileum tissue

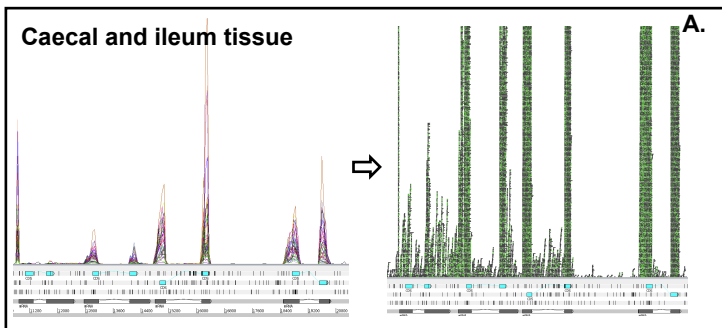

### Testis

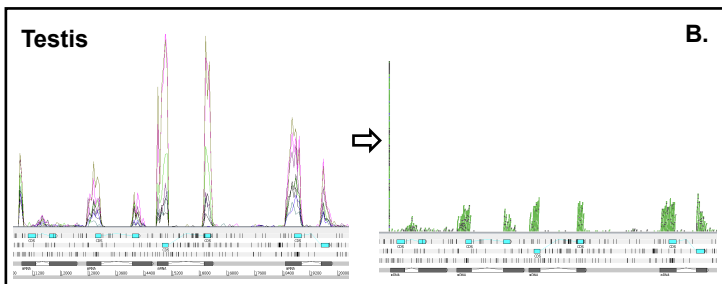

### Tibial bone tissue

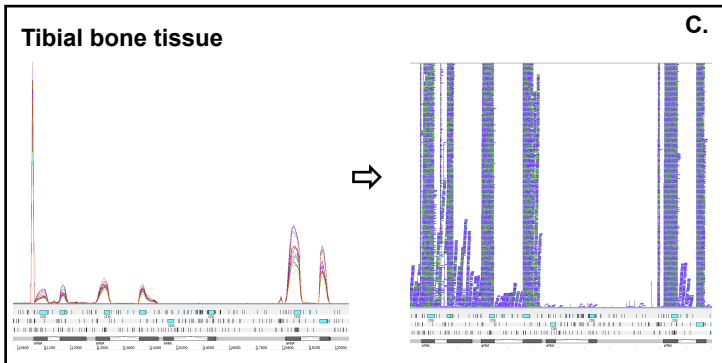

Supplement: Supplementary file 7 — A and B: RNA-seq data alignment of reads from caecal and ileum tissues (A) and testis (B) showing high coverage chIFITM1, not seen in the other studies analysed. C: RNA-seq data alignment of reads from bone tissue showing high coverage for chIFITM5. The figure focuses only on the 4 chIFITM transcripts showing on the left panel the coverage and on the right panel the stack view. The stack view of A and B also shows some distinct coverage for chIFITM5, more ordered than the other studies. (PDF 763 kb) [file 12864_2017_3801_MOESM7_ESM.pdf]
